# The role of the thymus in immunosenescence: lessons from the study of thymectomized individuals

**DOI:** 10.18632/aging.100122

**Published:** 2010-02-17

**Authors:** Victor Appay, Delphine Sauce, Martina Prelog

**Affiliations:** ^1^ Infections and Immunity, INSERM UMR S 945, Avenir Group, Hôpital Pitié-Salpêtrière, Université Pierre et Marie Curie-Paris6, Paris, France; ^2^ Department of Pediatrics, Pediatrics I, Medical University Innsbruck, Austria

**Keywords:** Thymus, T cells, immunosenescence, cytomegalovirus

## Abstract

The thymus is the major site of T cell production and a key organ of the immune system. Its natural involution during the course of life has cast doubts as to its importance for the integrity of our immunity in adulthood. We provide here an overview of the recent works focusing on the immunological evaluation of subjects thymectomized during early childhood due to cardiac surgery of congenital heart defects. These studies represent new advances in our appreciation of the role of the thymus in humans and more generally in our understanding of the development of immunosenescence.

## The role of the thymus

The thymus is a central lymphoid organ in humans. It is devoted to thymocyte differentiation and maturation, and is therefore the primary source of circulating T lymphocytes. Anatomically, the thymus is located in the upper anterior portion of the thorax, just behind the sternum and in front of the great vessels. Its weight in proportion to body weight is greatest shortly before birth. Although its size continues to increase until it reaches its maximum absolute weight during puberty, its functional compartments (the medulla and the cortex) and T cell output activity diminish after the first years of life onwards. Subsequently, the thymus undergoes a process known as involution, which is defined as a decrease in the size, weight and activity of the gland with advancing age. Thymic involution is thought to result from high levels of circulating sex hormones in particular during puberty, lower population of precursor cells from the bone marrow and changes in thymic microenvironment. Although it continues to serve as the site of T-cell differentiation and maturation throughout adulthood [1], the thymus largely degenerates into fatty tissue in elderly adults [2]. Since the atrophy of this organ and the reduction of its activity are natural processes which start relatively early in life, its importance beyond the initial production of T cells has remained a matter of debate and its role during adulthood has even be disregarded.

## Congenital heart defects and thymectomy

In order to facilitate the surgical correction of life-threatening congenital heart defects (CHD), it is common to ablate this gland, a process known as thymectomy. CHD is one of the most common defects at birth. The Children's Heart Foundation estimates that nearly one of every 100 babies is born with a CHD (http://www.childrensheartfoundation.org). CHD include a number of problems: septal defects, defects causing obstruction in the heart or blood vessels, cyanotic defects or even complex abnormalities. Some of these defects are mild and may need little or no medical treatment even through adulthood. But others can be lethal, either immediately to the newborn or over time. In this case, invasive surgery to correct the defect remains the solution of choice. However, surgical access to the heart and great vessels is obstructed by the thymus, particularly after birth or during infancy when it occupies the largest relative space. The thymus is thus removed to access the heart region in order to repair the defect. Over the last 30 years, open heart surgery in newborns has become an increasingly safe and routinely performed surgical procedure, in particular since there has been no clinical report of immune disorders (e.g. immunodeficiency) as a consequence of thymectomy in CHD subjects [3, 4]. In rodents, it has long been shown that thymectomy can result in partial immunodeficiency, mainly affecting cell mediated immune responses [5]. However, rodents are born with a relatively immature immune system and an imcomplete TCR repertoire, whereas human newborns should have already a fully developed TCR repertoire diversity at birth [6, 7]. Young adults thymectomized during early childhood represent nonetheless a particularly informative group to evaluate the importance of the thymus beyond the production of the initial T cell stock, and to study the long-term consequences of early thymectomy in adult life.

## Consequences on the T cell compartment

A number of studies have shown that thymectomy in infants results in alterations of the peripheral T cell compartment characterized by reduced CD4+ and CD8+ T cell counts, affecting mainly the naive T cells identified through the expression of CD45RA CD62L CCR7 and CD27 [8-14]. Decrease of naive CD4+ T cells was found to depend on chronological age and time post thymectomy [13]. Moreover, thymectomized individuals present decreased proportions of CD4+ T cells expressing CD31 or CD103, or displaying  T cell receptor excision circles (TREC) contents (i.e. markers of recent thymic emigrants)[9, 13, 14]. These alterations are attributed to the loss of thymic function, with an increase of peripheral proliferation of naive T cells. Analyses of the TCR repertoire revealed also a marked reduction in diversity within the CD8+ T cell compartment in some thymectomized patients, close to the changes found in individuals of old age, reflecting the accumulation of oligoclonal memory T cell populations in the periphery [14]. In a study with 20 cardiac transplants recipients, who had thymectomy 1 to 10 years prior to transplantation, a restricted TCR repertoire was also reported [12]. Regulatory CD4+CD25+ T cell numbers have been shown to be reduced in thymectomized patients, despite no apparent change in the proportions of the naive regulatory T cells (CD4+CD25+CD62L+) [11, 14]. It is possible that regulatory T cells mature earlier in life than other CD4+ T cells, and thus, may be less influenced by thymectomy. On the functional side, lymphocytes of thymectomized patients show an appropriate proliferative response to tetanus toxoid and phytohemagglutinin [8, 9]. Moreover, already existing memory T cells (e.g. specific for persisting viruses like EBV) in thymectomized patients present regular functional and phenotypic attributes; thus thymectomy does not seem to prevent the normal development of memory T cells [14]. Overall, these findings highlight a reduced production of naïve CD4+ and CD8+ T cells, and a disequilibrium of the naïve to memory T cell ratio, as consequences of thymectomy.

## Thymectomy and the heterogeneity of profiles

Importantly, studies on thymectomized individuals show also that there is a certain degree of heterogeneity between donors with regard to immunological attributes: some thymectomized individuals present satisfactory naïve T cell counts (i.e. close to non-thymectomized controls) despite no signs of thymic regeneration, while other donors present marked alterations. Despite their young age, some patients can have indeed profound perturbations that are typically associated with advanced aging (i.e. > 75 years old): decreased T cell count and very low naïve T cell frequency, reduced T cell repertoire diversity, and increased numbers of highly differentiated memory T cells with shortened telomeres [14]. Several factors may explain the heterogeneity between thymectomized patients in terms of immune phenotype and the maintenance of the peripheral naïve T cells in some donors. First, cervical extensions of the thymic tissue may remain *in situ* and contribute to the influx of naïve T cells; second, T cells may be generated *de novo* at extrathymic sites; third, human recent thymic emigrants generated before thymectomy may be long-lived and persist for decades; and fourth, antigen-independent homeostatic proliferation in the peripherymay compensate for the loss of thymic output in thymectomized patients. Nonetheless, extrinsic factors that impact indirectly on the level of naïve T cells and thus the function of the thymus may also be contemplated. Of note, a recent publication shows that exacerbated alterations in the T cell compartment in young adults thymectomized after birth were observed in those who were cytomegalovirus (CMV) seropositive [14]. Although an increased risk of acquiring CMV due to a potentially weakened immunity associated with thymectomy cannot be excluded, this marked immunosenescent phenotype is most likely the direct consequence of CMV infection through the establishment of an anti-CMV immune response in thymectomized patients. CMV is indeed known to impose a particular strong pressure on the immune system in normal healthy individuals [15]. CMV infection results in a massive expansion of CMV specific memory T cells, which can start from the early days of life and can reach up to 40% of total T cells during chronic infection [16]. Normal healthy adults infected with CMV present generally reduced proportions of naïve T cells and an accumulation of highly differentiated memory T cells associated with a loss of T cell repertoire diversity compared to CMV seronegative controls [17]. CMV infection is connected to the phenomenon of memory inflation, which is characterized by a progressive increase in the number of CMV specific memory T cells during chronic infection, with the continuous recruitment of naïve T cells, as shown in the murine CMV infection model [18]. In the context of inadequate T cell renewal due to thymectomy, CMV infection may thus lead to premature exhaustion of the naïve T cell compartment and loss of T cell repertoire diversity. Thymectomized individuals infected with CMV represent obviously an extreme situation, nonetheless its study provides interesting insights underlying the long term consequences of infections on our immune system and the development of immunosenescence with age. We learn that beyond its role in the initial production of T lymphocytes, the capacity of the thymus to produce T lymphocytes is necessary to maintain the integrity of the cellular immunity in the face of recurrent challenges by pathogens during the course of life, and thus to delay the onset of immunosenescence.

## Immune risk profile

Can thymectomy represent an immunological risk for CHD patients who underwent open heart surgery, in particular considering the high prevalence of CMV infection in the general population (50 to 80%)? To date, it is unclear whether thymectomized patients with a prematurely aged immune system are at greater risk to develop inflammatory diseases, autoimmunity, or cancer and may suffer from increased morbidity or mortality due infectious diseases and opportunistic pathogens, as this is observed with old age. Considering that some thymectomized patients present significant reductions in naïve T cell frequencies, immune responses to new antigens or vaccination may be diminished. In the elderly, poor responses to new infectious antigens and vaccinations have been explained by the reduction in recent thymic emigrants associated with immunosenescence [19]. Only one prospective cohort study analyzed the specific humoral immune response to a new antigen by immunizing thymectomized children with tick-borne encephalitis (TBE) vaccine [20]. The thymectomized children showed a significantly delayed primary immune response compared to age-matched, non-thymectomized children, similar to the findings of TBE vaccination in elderly patients after physiological thymus involution [21]. A decreased ability of thymectomized patients to respond appropriately to new antigens may gain more relevance in later life. It is important to bear in mind that the oldest thymectomized CHD patients are still young, since open heart surgery in newborns is a relatively recent surgical procedure (safely performed over the last 30-40 years). Follow-up programs (e.g. infection rates and antibody levels against vaccines) of thymectomized adults that reach older age will be required to establish if thymectomy represents a risk associated with higher than expected rates of age-associated immune conditions. It is likely that patients with residual thymic tissue after heart surgery, late thymectomy or CMV seronegativity will have close to normal immune attributes and will develop no related clinical conditions. However, one may speculate that early and complete resection of the thymus followed by infection with CMV may have adverse consequences on the immune competence in the long run. Decreased  T cell count,  very low naïve T cell frequency, reduced T cell receptor repertoire diversity, and increased numbers of senescent like memory T cells, as found in CMV infected thymectomized patients, are clear signs of immune deterioration. These alterations of the T cell compartment are reminiscent of the immune risk phenotype (IRP), defined by gerontologists as a cluster of immune measures that are predictive of early all-cause mortality in the elderly [22, 23]. In view of the immediate and obvious benefit of open heart surgery in the context of CHD, the adverse consequences of thymectomy may be considered as secondary. Nonetheless, partial resection of the thymus during cardiac surgery should be encouraged in order to limit premature aging of the immune system.
